# Atypical emphysema formation in a never-smoker with scleroderma-related interstitial pneumonia: a case report

**DOI:** 10.11604/pamj.2022.42.295.29442

**Published:** 2022-08-19

**Authors:** Rahma Ben Jazia, Jihene Ayachi, Raja Ghzel, Ameni Kacem, Iteb Ben Limem, Amira Faidi, Dhouha Ben Braiek, Farouk Chatbouri, Anis Maatallah

**Affiliations:** 1 Pulmonology Department, Ibn El Jazzar University Hospital, Kairouan, Tunisia,; 2 Medical Intensive Care Unit, Ibn El Jazzar University Hospital, Kairouan, Tunisia,; 3 Emergency Department, Ibn El Jazzar University Hospital, Kairouan, Tunisia

**Keywords:** Systemic sclerosis, combined-pulmonary emphysema, lung fibrosis, case report

## Abstract

Scleroderma is an autoimmune connective tissue disorder which is characterized by fibrosis of visceral organs, blood vessels and skin. The most common manifestations of lung disease in systemic sclerosis are interstitial lung disease and pulmonary hypertension and, together, are the leading cause of mortality in systemic sclerosis. Recently, we notice a new pattern called Combined-pulmonary emphysema and lung fibrosis. Most patients with this entity are male smokers or ex-smokers. This entity is characterized by the coexistence of both centro-lobular and para-septal emphysema in the upper lobes and interstitial lung disease in the lower lobes. Here, we present a case of a nonsmoker adult woman with systemic sclerosis, in which High Resolution Computed Tomography of lung showed combined fibrosis and emphysema with atypical radiological presentation and unusual distribution. This case outlines the importance of recognizing the presence of combined fibrosis and emphysema in patient with systemic sclerosis even without smoking history.

## Introduction

Interstitial Lung Disease (ILD) is a common finding of Systemic Sclerosis (SSc) mainly presenting in the form of Non-Specific Interstitial Pneumonia (NSIP) and deeply affecting patients' prognosis [1]. While ILD in SSc is well described, concomitant emphysema, or the so-called Combined-pulmonary emphysema and lung fibrosis (CPFE) is being increasingly recognized. Most patients with this CPFE are male smokers or ex-smokers. This entity is characterized by the presence of concomitant upper-lobe bullous emphysema, lower-lobe interstitial fibrosis. We herein report a case of coexistence of emphysema and fibrosis in a nonsmoker adult woman with SSc with atypical radiological presentation.

## Patient and observation

**Patient information:** a 51-year-old female patient was referred to Pulmonology Department for progressive onset of dyspnea on exertion evolving since two years and worsened last 2 months. No history of cough, wheeze, hemoptysis or fever was noticed. She had no history of chronic illnesses like hypertension or diabetes mellitus, and family history was unremarkable. She is non-smoker without significant environmental or occupational exposure. She is not married and had no children.

**Clinical findings:** on physical examination, her skin was smooth, tense, shiny and mask like. It was firm and could not be picked up. The patient exhibited stiffness in movements of the extremities. We also noticed that terminal phalanges were stiff, deformed and showed loss of flexibility. The patient was afebrile, she had blood pressure of 120/60mmHg, heart rate of 90 beats/min and respiratory rate of 26 breaths/min with an oxygen saturation of 89% on room air. Lung auscultation revealed dry crackles in the base of the right lung and was significant for abolished breathing sounds in the left hemi thorax.

**Figure 1:**
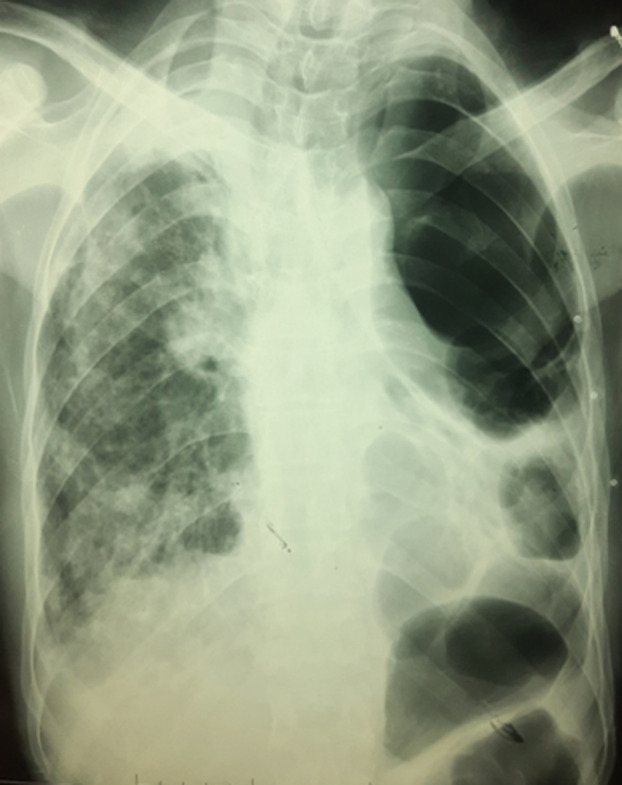
chest X-ray showing diffuse and severe emphysema in the left lung with mediastinal deviation and a reticular shadow in the right lung

**Figure 2:**
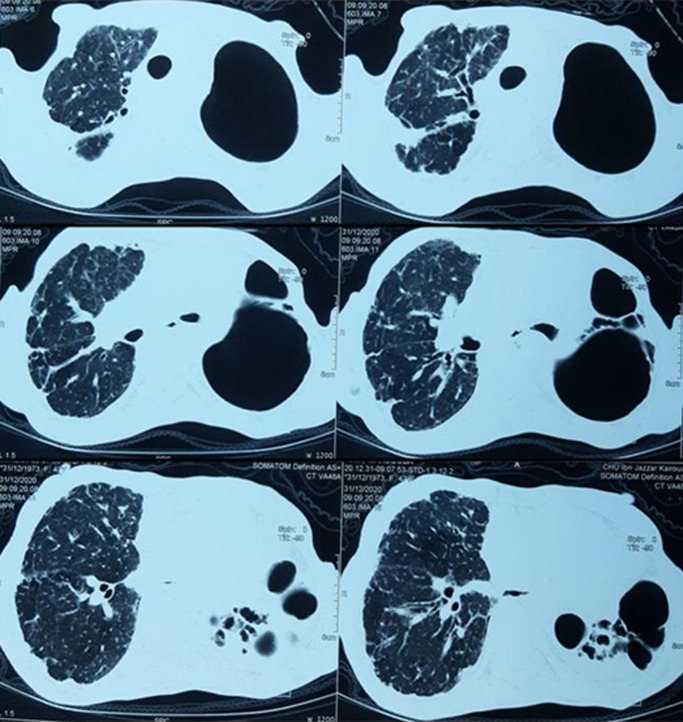
thoracic computed tomography (CT) scan showing extensive and severe emphysema bullae in the left lung with traction bronchiectasis and reticulation with volume loss in the right lung

**Diagnostic assessment:** laboratory results showed: positive antinuclear antibodies against centromere B, Scl-70, and Ro-52; normocytic normochromic anemia (Hemoglobin at 9 gr/dl), without biological inflammatory syndrome. Electrocardiography (ECG) showed normal sinus rhythm with tachycardia of 100 beats/min. A Chest X-ray (CXR) evidenced diffuse and huge emphysema in the left lung, with mediastinal deviation and diffuse reticular shadow in the right lung (Figure 1). A thoracic High-resolution computed tomography (HRCT) was performed showing extensive and severe emphysema bullae in the left lung and traction bronchiectasis combined with interstitial lung disease in the right lung (Figure 2). In front of this huge emphysema in a nonsmoker woman, alpha-1 antitrypsin (AAT) test was performed and AAT deficiency was excluded. Arterial blood gas (ABG) at room air revealed significant hypoxemia (PaO_2_: 54mmHg) (normal range: 70-100mmHg) with hypercapnia (PaCO_2_: 51mmHg) (normal range: 38-42mmHg) and respiratory acidosis (pH: 7.36) (normal range: 7.38-7.42).

During oxygen supplementation in the department, we observed the increase in paO_2_ to 72 mmHg under 3 L/min of oxygen and the normalization of other blood gas parameters. Lung function testing revealed severe restrictive impairment (forced vital capacity (FVC) of 1.10 L and 49% of predicted). The total lung capacity (TLC) was reduced to 2,3 L, and 59% of predicted, with decreased diffusion capacity for carbon monoxide (DLCO) (6.21 mL/min/mm Hg and 49.4% of predicted). A transthoracic echocardiography was performed, showing normal left ventricular wall motion with elevated pulmonary artery systolic pressure (PASP) of 51mmHg consistent with severe pulmonary arterial hypertension (PAH).

**Diagnosis:** in this case, clinical, radiological and laboratory features were consistent with a diagnosis of SSc with chronic respiratory failure caused by CPFE in a nonsmoker adult woman with atypical radiological presentation.

**Therapeutic interventions:** only home oxygen therapy was administrated. Corticosteroids and immunosuppressive drugs were not able to be administered in this case in front of the presence of chronic respiratory failure. The evolution was marked by the death of the patient 1 month after discharge from the hospital.

### Consent of patient

The patient had provided verbal consent to publish this report since we wrote the first version of the manuscript. We had clearly explained the scientific importance to publish this case and patient was reassured about the guaranteed anonyma.

## Discussion

SSc is a disease characterized by autoimmunity, vasculopathy and fibrosis. The effects are wide-ranging, affecting multiple different organs. ILD and pulmonary hypertension are the most common manifestations of lung disease in SSc and, together, are the leading cause of mortality in SSc [1]. The pathogenesis is complex and only partially understood. Initial insults that precede fibrosis include alveolar epithelial cell and endothelial cell injuries, which induce the release of fibroblast activating mediators. Fibroblasts differentiate into myofibroblasts, which results in accumulation of extracellular matrix components and collagen, leading to fibrosis [2]. The most common pattern of ILD on imaging is NSIP [3,4]. While ILD in SSc is well described, concomitant emphysema and pulmonary fibrosis is being increasingly recognized, even in those with little to no smoking history. In 2005, Cottin *et al*. described for the first time the defined syndrome termed “combined pulmonary fibrosis and emphysema (CPFE)” [5]. The clinical characteristics of CPFE are: (A) coexistence of emphysema predominantly in the upper lungs and fibrosis predominantly in the lower lungs, exhibited in HRCT; (B) normal spirometric values and lung volumes despite extensive lung disease, as well as marked impairment in gas exchange; (C) often complicated with the presence of pulmonary hypertension (PH) suggesting a poor prognosis; and (D) a risk of lung cancer [5].

When CPFE was first described by Cottin *et al*., patients with Connective tissue disease (CTD)-associated ILD were excluded from the study [5]. However, in 2011, Cottin et al published a multi-center, retrospective review of patients with CTD-related ILD and concomitant emphysema on HRCT, mainly among smokers or former smokers with rheumatoid arthritis and SSc [6]. Patients with CTD-associated CPFE are more likely to be women, significantly younger, and tend to have less severe outcomes than their idiopathic CPFE counterparts. This cohort of 34 patients was observed to have paraseptal emphysema in 62% and centrilobular emphysema in 71%. On HRCT, centrilobular emphysema appears as small areas of low attenuation without a perceptible wall, containing a ‘white dot’ centrilobular artery in its center, which are surrounded by normal attenuation parenchyma. Typically, it has upper lobe predominance [6]. Antoniou *et al*. described a cohort of patients with SSc- ILD and found that 41 of 333 patients (12.3%) had CPFE [7]. Interestingly, 15 of these 41 patients (7.5%) were never-smokers. Among the never-smoking CPFE patients, there was an increased prevalence in men, and they also had a greater impairment in gas exchange. The extent of emphysema was greater in the upper zones compared to the lower zones, and the extent of ILD was not linked to the extent or the proportion in the upper zone [7].

We herein described novel findings different from this previous report. Indeed, contrary to previous report that indicated the presence of concomitant emphysema in the upper lobes and pulmonary fibrosis in the lower lung zones as the radiological findings [5-7], the distribution of emphysema in this case was unusual. The patient´s HRCT imaging demonstrated diffuse, unilateral and severe panlobular emphysema with destruction of the entire left lung with ILD in the right lung. However, the mechanism of emphysema formation in SSc-ILD has never been clarified. Recently, we suggested that peripheral vasculopathy frequently seen in SSc patients might occur that can result in destruction of the fibrously thickened alveolar walls, resulting in the emphysematous change seen in SSc-ILD. In this case, the patient has no history of active or passive smoking. However, CPFE is reported to have a strong correlation with cigarette smoking, and most patients present with approximately 40-pack-year histories or more [6]. Cottin et al reported four CPFE patients with CTD that were nonsmokers, including two with SSc [6]. In addition, a young woman with CPFE, a nonsmoker, was reported to have a surfactant protein-C (CFTPC) mutation [8]. Therefore, CPFE may have an underlying genetic predisposition.

CPFE has a characteristic pulmonary function feature different from pure emphysema, which is characterized by the unexpected relatively normal lung volumes contrasted by a severely reduced diffusing capacity. In many research, mean values of FVC and TLC in CPFE are usually within relatively normal range, whereas DLCO is severely diminished [9]. The preserved lung volumes may be attributed to the counterbalanced effects of the hyperinflation defect of emphysema and the restrictive defect of pulmonary fibrosis. In this case, we found that pulmonary function feature is different from those in other report. Indeed, our patient had severe restrictive impairment with reduced total lung capacity and severe hypoxemia with respiratory acidosis and hypercapnia. The reduced diffusing capacity in this case, such us other report, may be due to the overlapping negative effects of both emphysema and pulmonary fibrosis on the gas exchange.

## Conclusion

CPFE is a distinct, still under-recognized, pulmonary complication of SSc. We report an atypical radiological presentation of CPFE in a nonsmoker woman. The existence of emphysema in nonsmokers with SSc-related ILD provides indirect support for a pathogenetic basis for the CPFE syndrome.
